# Effectiveness of pharmacist-led medication reconciliation on medication errors at hospital discharge and healthcare utilization in the next 30 days: a pragmatic clinical trial

**DOI:** 10.3389/fphar.2024.1377781

**Published:** 2024-03-28

**Authors:** Maja Jošt, Mojca Kerec Kos, Mitja Kos, Lea Knez

**Affiliations:** ^1^ University Clinic Golnik, Golnik, Slovenia; ^2^ University of Ljubljana, Faculty of Pharmacy, Ljubljana, Slovenia

**Keywords:** medication reconciliation, medication error, healthcare utilization, transitions of care, pharmacist, patient discharge, safety

## Abstract

Transitions of care often lead to medication errors and unnecessary healthcare utilization. Medication reconciliation has been repeatedly shown to reduce this risk. However, the great majority of evidence is limited to the provision of medication reconciliation within clinical trials and countries with well-established clinical pharmacy. Thus, this pragmatic, prospective, controlled trial evaluated the effectiveness of routine pharmacist-led medication reconciliation compared to standard care on medication errors and unplanned healthcare utilization in adult general medical patients hospitalized in a teaching hospital in Slovenia. All patients hospitalized in a ward where medication reconciliation was integrated into routine clinical practice were included in the intervention group and received admission and discharge medication reconciliation, coupled with patient counselling. The control group consisted of randomly selected patients from the remaining medical wards. The primary study outcome was unplanned healthcare utilization within 30 days of discharge, and the secondary outcomes were clinically important medication errors at hospital discharge and serious unplanned healthcare utilization within 30 days of discharge. Overall, 414 patients (53.4% male, median 71 years) were included—225 in the intervention group and 189 in the control group. In the intervention group, the number of patients with clinically important medication errors at discharge was significantly lower (intervention vs control group: 9.3% vs 61.9%). Multiple logistic regression revealed that medication reconciliation reduced the likelihood of a clinically important medication error by 20-fold, while a higher number of medications on admission was associated with an increased likelihood. However, no significant differences were noted in any and serious unplanned healthcare utilization (intervention vs control group: 33.9% vs 27.8% and 20.3% vs 14.6%, respectively). The likelihood of serious healthcare utilization increased with the age of the patient, the number of medications on admission and being hospitalized for an acute medical condition. Our pragmatic trial confirmed that medication reconciliation, even when performed as part of routine clinical practice, led to a substantial reduction in the risk of clinically important medication errors at hospital discharge but not to a reduction in healthcare utilization. Medication reconciliation is a fundamental, albeit not sufficient, element to ensure patient safety after hospital discharge.

**Clinical Trial Registration:** https://clinicaltrials.gov/search?id=NCT06207500, identifier NCT06207500

## 1 Introduction

Hospitalization is a stressful event in a patient’s life that poses patients to a generalized risk of adverse health events during hospitalization and after hospital discharge (Krumholz, 2013). Medication errors at transitions of care, often represented by unintentional medication discrepancies, contribute considerably to this risk (Uitvlugt et al., 2022). Unintentional discrepancies occur in approximately half of hospitalized patients upon hospital admission (Cornish et al., 2005; Tam, 2005; Hellström et al., 2012), and they persist to a similar extent at hospital discharge (Knez et al., 2011b; Grimes et al., 2011). Most importantly, medication errors at transitions of care can lead to patient harm. Namely, unintentional discrepancies at hospital admission resulted in an adverse drug event (ADE) in one-fifth of cases even during a short hospital stay (Jošt et al., 2022). Furthermore, discrepancies at hospital discharge, regardless of their intent, were often associated not only with ADEs but also with increased healthcare utilization and hospital readmission (Coleman et al., 2005; Forster et al., 2005; Uitvlugt et al., 2022). Many of these events are preventable.

Medication reconciliation has been introduced to improve patient safety at transitions of care. Medication reconciliation is the process of identifying an accurate list of a person’s current medicines and comparing it with the current list in use, identifying any discrepancies, and documenting any changes, thereby resulting in a complete list of medicines, accurately communicated (Anon, 2011). Indeed, medication reconciliation has been repeatedly shown to reduce medication errors at transitions of care, while its impact on more patient-centered outcomes has led to mixed results, also in several well-designed studies (Cebron Zerovnik, and Kos 2019; Cheema et al., 2018; Ensing et al., 2015; Michaelsen et al., 2015; Mueller et al., 2012). While some studies on medication reconciliation performed in isolation or as part of more complex interventions at transitions of care have shown that post-discharge all-cause and medication-related healthcare utilization were substantially reduced (Gillespie et al., 2009; Marusic et al., 2013; Lenssen et al., 2018; Ravn-Nielsen et al., 2018; Snyder et al., 2020; Schnipper et al., 2022), others have not (Phatak et al., 2016; Karapinar-Çarkıt et al., 2019; Lea et al., 2020; Ceschi et al., 2021; Gurwitz et al., 2021; Kempen et al., 2021; Johansen et al., 2022).

Heterogeneity in the outcomes of medication reconciliation studies is expected. Indeed, there is wide variability in the interventions performed, ranging from providing medication reconciliation only at hospital admission and/or discharge to upgrading it with medication review and various post-discharge interventions, such as patient and caregiver engagement through phone calls or post-discharge visits and communication with primary care physicians, pharmacists and nurses (Dautzenberg et al., 2021). In addition, implementing complex interventions such as medication reconciliation is challenging (Gesell et al., 2021). Many factors contribute to successful implementation, and if these factors are not adequately addressed, it may affect the final outcomes (Jošt et al., 2022; Schnipper et al., 2022). Therefore, medication reconciliation should be tailored to the specifics of each healthcare facility, which inherently limits the generalizability of study results. In facilities with limited clinical pharmacy activities, which include those in many Central-Eastern European countries, previously unreported barriers may be present (Režonja et al., 2010; Knez et al., 2011b; Urbańczyk et al., 2023). In addition, the sustainability of an intervention delivered in the tightly controlled environment of a clinical trial is not guaranteed when the intervention is transferred to everyday clinical practice (Ford and Norrie, 2016; Gesell et al., 2021).

The aim of our pragmatic trial involving hospitalized adult medical patients was to evaluate the impact of routine pharmacist-led medication reconciliation on the occurrence of clinically important medication errors at hospital discharge and healthcare utilization within 30 days after hospital discharge.

## 2 Methods

### 2.1 Study design and participants

A pragmatic, prospective, controlled clinical trial was conducted in hospitalized adult medical patients. Patients in the intervention group were offered a pharmacist-led medication reconciliation service, while patients in the control group received standard care.

This study was conducted in five general medical wards at the University Clinic of Respiratory and Allergic Diseases Golnik in Slovenia. The patients admitted to this clinic belong to the population of general medical patients, who are most frequently admitted due to acute pulmonary and cardiovascular disorders or diagnostics in pulmonary diseases. Patients were assigned to the intervention or control group according to their admission ward: one ward, where medication reconciliation was implemented in routine clinical practice, served as the *intervention ward*, whereas the remaining four wards served as *control wards*. Despite no formal randomization into the intervention or control group, the patients’ ward allocation was random, as it depended primarily on bed availability and was thus not influenced by the conduct of this study. All patients admitted to the intervention ward were included in the intervention group, while in the control group, patients were randomly selected from among all patients, admitted to the four control wards using Research Randomizer (Urbaniak and Scott, 2011), and followed the temporal dynamics of patient inclusion in the intervention group. Our aim was to include an equal number of patients in both groups.

All adult general medical patients admitted to the study wards were eligible to participate in this study, except those who did not speak Slovenian, were transferred from another ward or were previously included in the same study. Patients who were hospitalized only for diagnostic purposes, patients transferred to another ward or hospital, patients who died during hospitalization, and patients from the control group who were offered medication reconciliation were subsequently excluded from this study. Because of the study design, participants and ward staff were not blinded to treatment assignment.

All procedures performed in this study were conducted in accordance with the ethical standards of the institutional and national research committee (National Medical Ethics Committee in Slovenia, protocol number 0120-223/2019/4) and with the 1975 Declaration of Helsinki and its subsequent amendments or comparable ethical standards. Informed consent was obtained from all patients included in this study. This study is registered at ClinicalTrials.gov (NCT06207500).

### 2.2 Intervention

Patients in the intervention group were offered a pharmacist-led medication reconciliation service. The service was provided by clinical pharmacists or final year residents in clinical pharmacy working under the supervision of a clinical pharmacist. The service included medication reconciliation at hospital admission and at discharge, coupled with patient counselling. To guarantee uniform execution of the intervention, a standard operating procedure was created, the pharmacists were trained accordingly, and peer-to-peer supervision was performed prior to the start of this study. In brief, at hospital admission and after reviewing all available medical and pharmacy records, the best possible medication history (BPMH) was obtained through interviews with the patients or caregivers. The BPMH was compared with in-hospital therapy to identify discrepancies. All discrepancies were discussed with the treating physician, unintentional discrepancies were reconciled, and intentional discrepancies were documented in the medical records. At hospital discharge, medication reconciliation was performed to ensure that all unintentional discrepancies between a patient’s BPMH and discharge medicines were reconciled and that all intentional discrepancies were explained in the discharge letter. In addition, face-to-face patient counselling on discharge medicines and aligned changes was conducted and coupled with written instructions in lay language. At every step, clinical pharmacists worked in close collaboration with the treating physicians, and all the documentation was prepared by the clinical pharmacist and approved by the physician. All the relevant documents were included in the patients’ medical records.

Patients in the control group received only written instructions on discharge medicines in the discharge letter, according to standard practice. Patients in both groups may have received clinical pharmacy services such as therapeutic drug monitoring services, medicine’s adjustments in poor renal function, and drug interaction assessments.

### 2.3 Data collection and outcome assessment

Data collection and outcome assessment were performed by research pharmacists, who were clinical pharmacists or final year residents in clinical pharmacy and were not involved in the treatment of the included patients. To ensure standardized data collection and outcome assessment, a standard operating procedure was established, and the pharmacists were trained accordingly before starting their collaboration. In case of uncertainties in outcome assessment, the research pharmacists consulted with each other to reach a consensus.

The data were collected from patients’ medical records and study documentation. Patient’s comorbidities were assessed by the Charlson Comorbidity Index (Quan et al., 2005; Shebeshi, Dolja-Gore, and Byles, 2021). The reason for a patient’s index hospital admission was retrieved from the discharge letter and grouped into acute or planned admissions. For patients in the control group, a BPMH was collected in the same way as in the intervention group. However, the BPMH served only for study purposes and was thus not documented in patients’ medical records.

To identify discrepancies at admission, the BPMH was compared to the medication data in the admission documentation. Likewise, to identify discrepancies at hospital discharge, the BPMH was compared to the discharge therapy. After reviewing the complete medical documentation related to the index hospitalization, discrepancies were classified as unintentional, undocumented intentional or documented intentional. A discrepancy was classified as *unintentional* if no medical reason was evident for the undertaken change in therapy, as *undocumented intentional* if a medical reason for the undertaken change in therapy was evident but not documented in the discharge letter, or *documented intentional* if the medical reason for the undertaken change in therapy was evident and documented in the discharge letter. Unintentional discrepancies and undocumented intentional discrepancies were defined as *medication errors,* and their clinical importance was assessed using a 4-point Likert scale ranging from not important, not very important, and very important to life-threatening medication errors. Very important and life-threatening medication errors represented *clinically important medication errors*.

For both groups, data on healthcare utilization up to 30 (±5) days after hospital discharge were collected through patient or caregiver phone interviews. Healthcare visits within 30 days of hospital discharge were defined as any visit to a general practitioner, specialist, emergency department (ED), or hospitalization. These visits were classified as unplanned if sudden health problems required medical attention and planned if the visits were scheduled. Data on mortality due to any reason were also collected 30 days after discharge. For each patient, only the most detrimental outcome was classified.

The primary study outcome was unplanned healthcare utilization, defined as the occurrence of any unplanned visits or death within 30 days from hospital discharge. Secondary outcomes included the occurrence of clinically important medication errors at discharge and serious unplanned healthcare utilization, defined as the occurrence of any unplanned ED visit, hospitalization or death within 30 days from hospital discharge.

### 2.4 Sample size calculation and statistical analysis

The literature indicates that between 18% and 67% of patients make an unplanned visit to a healthcare facility within 1 month after hospital discharge (Al-Rashed et al., 2002; Marusic et al., 2013; Snyder et al., 2020; Graebek 2018). Based on the assumption that 30% of the individuals in the control group would require an unplanned healthcare visit, a sample size of 400 patients per group was considered necessary to observe a 30% reduction in these unplanned visits. This calculation assumed a statistical power of 80% and a significance level (α) of 0.05 and took into consideration a potential dropout rate of 10%.

Descriptive statistics were used to describe the baseline characteristics of the participants. A univariable statistical analysis was first performed to compare the intervention and control groups. The chi-square test or Fisher’s exact probability test was used for categorical variables, and the nonparametric Mann‒Whitney U test was used for continuous variables. Multiple logistic regression models were employed to examine the impact of pharmacist-led medication reconciliation on primary and secondary outcomes. The following covariates were used as potential predictors in the analysis: gender, age, number of medications before admission, comorbidities, type of and reason for admission, and duration of hospitalization. Prior to logistic regression, we ensured that the data met the necessary assumptions for the analysis, including the absence of multicollinearity by using a correlation matrix and variance inflation factor (VIF) methods among predictors. Model fit was evaluated using the Hosmer–Lemeshow goodness-of-fit test. Nagelkerke’s R^2^ was used to get an insight into the model’s explanatory power. The significance of individual variables was analyzed by the Wald statistical test. The results are presented as odds ratios (ORs) with 95% confidence intervals (CIs). All the statistical analyses were performed using the statistical program IBM SPSS Statistics version 28.0. A significance level of α = 0.05 was used for all tests.

## 3 Results

**FIGURE 1:**
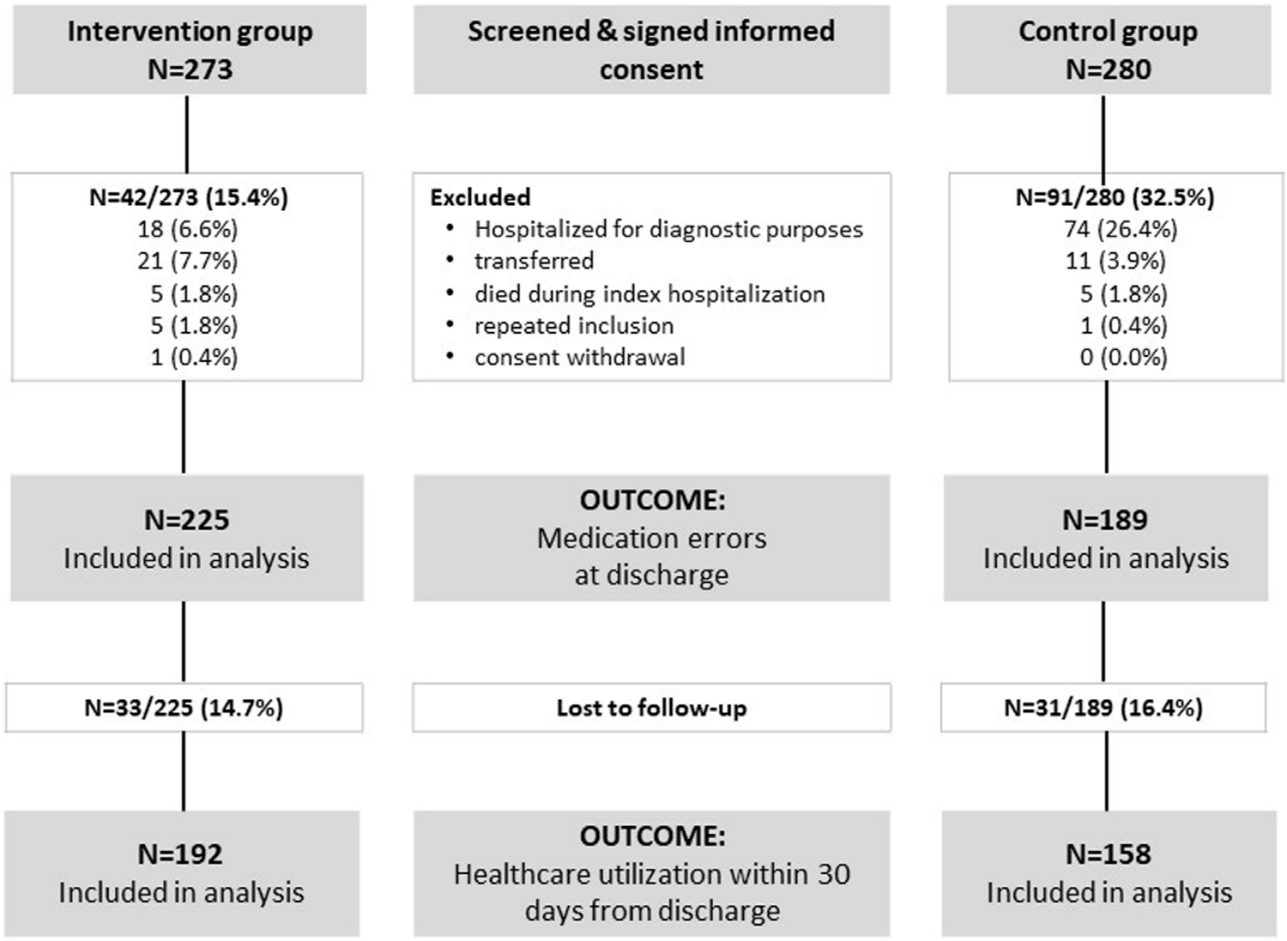
Study flow diagram.

A total of 553 patients were screened and agreed to participate in this study—273 in the intervention group and 280 in the control group. Some patients were subsequently excluded due to reasons arising after hospital admission, resulting in 414 patients remaining for further analysis—225 in the intervention group and 189 in the control group (Figure 1).

The included patients were evenly distributed between genders (53.4% male), most were of older age, with a median age of 71 years (interquartile range (IQR) 63-80), patients had a median Charlson Comorbidity Index of 2 (IQR 1-4) and a median intake of 7 medications on admission (IQR 4-10). There were no differences between groups (Table 1). In the intervention group, more patients (89.8%) were admitted for an acute health condition than in the control group (60.8%; *p* < 0.001). Additionally, the reason for admission differed between the groups, with more patients in the intervention group admitted due to infection and heart disease (*p* < 0.001). The length of hospital stay was shorter in the intervention group (6 days vs 7 days; *p* < 0.001).

**TABLE 1:** Patients’ baseline characteristics.

	All patients		Intervention group		Control group				*p-value*
	N = 414		N = 225		N = 189				
Gender; male (n, %)	221	53.4%	128	56.9%	93		49.2%		0.119*
Age (years; median, IQR)	71	(63–80)	72	(64–81)	70		(61–78)		0.063 **
Charlson Comorbidity Index (median, IQR)	2	(1–4)	2	(1–3)	2		(1–4)		0.265 **
Number of medications on admission (median, IQR)	7	(4–10)	7	(4–10)	7		(4–10)		0.680 **
Discrepancies on admission (n, %)	358	86.5%	191	84.9%	167		88.4%		0.304*
Admission type; acute (n, %)	317	76.6%	202	89.8%	115		60.8%		<0.001*
Reason for admission (n, %)									
Infection	125	30.2%	87	38.7%		38		20.1%	<0.001*
Respiratory disease	112	27.1%	42	18.7%		70		37.0%	
Heart disease	74	17.9%	46	20.4%		28		14.8%	
Malignancy	58	14.0%	24	10.7%		34		18.0%	
Other	45	10.9%	26	11.6%		19		10.1%	
Duration of hospitalization (days; median, IQR)	7	4%–10%	6	4–9		7		6–11	<0.001**

Abbreviations: IQR, interquartile range; significant *p* values are marked in bold.

*Chi square test; ** Mann‒Whitney U test.

### 3.1 Medication errors at hospital discharge

The majority of patients (97.8%) had at least one discrepancy at hospital discharge, with no difference between the intervention and control groups (Table 2). However, in the intervention group, significantly fewer patients had at least one medication error at discharge (75/225; 33.3% vs 181/189; 95.8%; *p* < 0.001), with a significantly lower number of medication errors per patient than in the control group (median 0, IQR 0-1 vs median 3; IQR 2-6; *p* < 0.001). Most importantly, a significant difference was also observed for clinically important medication errors, with 9.3% (21/225) of patients in the intervention group and 61.9% (117/189) in the control group (*p* < 0.001) having these types of errors. Some examples of clinically important medication errors are presented in Supplementary Table S1. Patients in the intervention group had significantly fewer clinically important medication errors per patient (median 0, IQR 0-0) than patients in the control group did (median 1, IQR 1-2; *p* < 0.001). Furthermore, the intervention considerably reduced the risk of clinically important medication errors by 20-fold, as shown by the multiple logistic regression model (OR 0.050, 95% CI 0.027–0.095; *p* < 0.001; Table 3). In contrast, the number of medications on admission had the opposite effect, albeit to a lesser extent (OR 1.173, 95% CI 1.092–1.259; *p* < 0.001).

**TABLE 2:** Medication errors and healthcare utilization at hospital discharge and within 30 days from discharge.

	All patients		Intervention group		Control group		*p-value*
Patients with discrepancies at discharge	N = 414		N = 225		N = 189		
Any discrepancy (n, %)	405	97.8%	218	96.9%	187	98.9%	0.154 *
Medication error (n, %)	256	61.8%	75	33.3%	181	95.8%	<0.001 *
Clinically important medication error (n, %)	138	33.3%	21	9.3%	117	61.9%	<0.001 *
Discrepancies per patient at discharge							
Any discrepancy; median (IQR)	4	(2–6)	3	(2–5)	5	(3–8)	<0.001 **
Medication error; median (IQR)	1	(0–3)	0	(0–1)	3	(2–6)	<0.001 **
Clinically important medication error; median (IQR)	0	(0–1)	0	(0–0)	1	(1–2)	<0.001 **
Healthcare utilization within 30 days from discharge	N = 350		N = 192		N = 158		
Unplanned healthcare utilization (n, %)	109	31.1%	65	33.9%	44	27.8%	0.227 *
Serious unplanned healthcare utilization (n, %)	62	17.7%	39	20.3%	23	14.6%	0.160 *
• Death^a^	16	4.6%	10	5.2%	6	3.8%	
• Hospitalization^a^	32	8.9%	17	8.9%	14	8.9%	
• Emergency department visit^a^	15	4.3%	12	6.3%	3	1.9%	

^a^ Most severe outcome.

*Chi square test; ** Mann‒Whitney U test.

Significant *p* values are marked in bold.

**TABLE 3:** Multiple logistic regression results.

Covariates	Outcome					
	Clinically important medication error at discharge		Unplanned healthcare utilization within 30 days of discharge		Serious unplanned healthcare utilization within 30 days of discharge	
	Nagelkerke’s R^2^ = 0.465; *P* = 0.535*		Nagelkerke’s R^2^ = 0.087; *P* = 0.119*		Nagelkerke’s R^2^ = 0.209; *P* = 0.740*	
	OR (95% CI)	*P value*	OR (95% CI)	*P value*	OR (95% CI)	*P value*
Gender (male vs. female)						
Female	Reference					
Male	0.969 (0.563-1.666)	0.909	1.282 (0.783-2.100)	0.323	1.555 (0.827–2.925)	0.171
Study group						
Control group	Reference					
Intervention group	0.050 (0.027–0.095)	**<0.001**	1.324 (0.791–2.216)	0.285	1.464 (0.762–2.815)	0.253
Age (years)	0.996 (0.973–1.019)	0.737	1.013 (0.993–1.034)	0.208	1.033 (1.003–1.064)	**0.031**
Charlson Comorbidity Index	1.044 (0.891–1.223)	0.596	1.106 (0.954–1.281)	0.183	1.188 (0.999–1.413)	0.052
Number of medications on admission	1.173 (1.092–1.259)	**<0.001**	1.049 (0.987–1.116)	0.125	1.102 (1.020–1.190)	**0.013**
Admission type						
Planned	Reference					
Acute	1.056 (0.545–2.048)	0.871	1.889 (0.918–3.888)	0.084	3.106 (1.133–8.517)	**0.028**
Reason for admission		0.314		0.874		0.300
Infection	Reference					
Malignancy	0.959 (0.356–2.584)	0.937	0.801 (0.338–1.896)	0.614	1.628 (0.486–5.456)	0.429
Heart disease	1.635 (0.475–5.632)	0.436	0.972 (0.301–3.140)	0.962	3.397 (0.729–15.835)	0.119
Respiratory disease	1.985 (0.700–5.627)	0.197	0.868 (0.338–2.226)	0.768	0.859 (0.219–3.371)	0.827
Other	0.926 (0.362–1.059)	0.881	1.141 (0.478–2.720)	0.766	1.488 (0.420–5.269)	0.538
Duration of hospitalization (days)	1.020 (0.983–1.059)	0.290	1.027 (0.993–1.063)	0.118	1.026 (0.986–1.067)	0.202

Abbreviations: OR; odds ratio; CI; confidence interval

Significant p values are marked in bold. *Hosmer-Lemeshow test

### 3.2 Healthcare utilization within 30 days from hospital discharge

Overall, unplanned healthcare utilization within 30 days of discharge was noted in approximately one-third of patients, with no significant differences between the intervention (65/192: 33.9%) and the control (44/158: 27.8%) groups (*p* = 0.227; Table 2). Serious unplanned healthcare utilization occurred in 20.3% (39/192) of patients in the intervention group and in 14.6% (23/158) of patients in the control group, with no difference between groups (*p* = 0.160). Unplanned hospitalizations occurred in 8.9% of patients in both groups, while 5.2% (10/192) and 3.8% (6/158) of patients died in the intervention and control groups, respectively.

According to the multiple logistic regression model, no significant associations were found between the intervention and other variables with unplanned healthcare utilization within 30 days from discharge. On the other hand, serious unplanned healthcare utilization was associated with increasing age (OR 1.033, 95% CI 1.003–1.064; *p* = 0.031), a greater number of medications on admission (OR 1.102, 95% CI 1.020–1.190; *p* = 0.013) and admission for an acute health condition (OR 3.106, 95% CI 1.133–8.517; *p* = 0.028), while the intervention had no significant effect (Table 3).

## 4 Discussion

The current pragmatic, prospective clinical trial in adult medical patients described a remarkable, 20-fold reduction in the risk of clinically important medication errors at hospital discharge through the provision of pharmacist-led medication reconciliation within routine clinical practice. However, our study was unable to demonstrate that this improvement translated into a reduction in unplanned healthcare utilization within the first month of hospital discharge.

The 414 included patients were older aged (median age >70 years), had comorbidities (median Charlson Comorbidity Index score of 2) and were treated with polypharmacy (median of 7 medications on admission), with no differences between groups. However, significantly more patients in the intervention group (89.8%) were hospitalized for an acute health condition, most commonly for infection or heart disease, than in the control group (60.8%). Despite the broad inclusion criteria of the present study, the included patients were representative of the population at high risk for medication errors, rehospitalization and mortality (Gleason et al., 2010; Allaudeen et al., 2011; van Walraven, 2014; Alassaad et al., 2015).

As expected, almost all patients experienced a change in pharmacotherapy at hospital discharge, exposing them to a risk of discrepancies. Patients who underwent medication reconciliation had a significantly lower median number of discrepancies (intervention vs control group: 3 vs 5), medication errors (intervention vs control group: 0 vs 3) and clinically important medication errors (intervention vs control group: 0 vs 1; all *p* < 0.001), all of which were assessed by independent observers. Moreover, the proportion of patients with at least one clinically important medication error at hospital discharge was 6-fold lower in the intervention group (9.3%) than in the control group (61.9%; *p* < 0.001), and, after adjustment for other patient and hospitalization characteristics, patients in the intervention group benefited from a 20-fold reduction in the risk of a clinically important medication error (multiple logistic regression, Table 3). Our results are consistent with those of previous studies that have repeatedly shown that pharmacist-led interventions reduce medication discrepancies and medication errors at transitions of care and are among the ones showing the greatest impact (Nickerson et al., 2005; Mueller et al., 2012; Ensing et al., 2015; Michaelsen et al., 2015; Cheema et al., 2018; Lipovec et al., 2019).

However, the reduction in medication errors at discharge was not accompanied by decreased healthcare utilization. Approximately one-third of patients had an unplanned healthcare visit or died within 30 days of discharge; there was no significant difference between the intervention group and the control group (33.9% vs 27.8%, *p* > 0.05), and there was no association with other patient or hospitalization characteristics (multiple logistic regression, Table 3). Moreover, there were no differences between the groups in terms of serious unplanned healthcare utilization, including ED visits, hospitalization or death (intervention vs control group: 20.3% vs 14.6%, *p* > 0.05); however, the likelihood of serious unplanned healthcare utilization increased with patient age, number of medications at admission and being hospitalized hospitalization for an acute medical condition (multiple logistic regression, Table 3).

Patient enrolment began in October 2019. However, it was temporarily halted due to the declaration of the coronavirus disease 2019 (COVID-19) epidemic in Slovenia on 12 March 2020 and finally ended with the second declaration of the epidemic on 18 October 2020, after inclusion of only 50% of the planned patients. Consequently, the planned sample size of 800 patients was not reached, which led this study to be underpowered to detect the initially expected difference in unplanned healthcare utilization. However, the current results do not indicate a difference between the groups, and our observations would probably not be affected by increasing the sample size. In some studies, pharmacist-led medication reconciliation reduced healthcare utilization, prehospitalization or ED visits and other outcomes (Gillespie et al., 2009; Marusic et al., 2013; Ravn-Nielsen et al., 2018; Snyder et al., 2020), while our study is one of numerous others that failed to demonstrate this (Graabaek et al., 2019; Karapinar-Çarkıt et al., 2019; Lea et al., 2020; Ceschi et al., 2021; Kempen et al., 2021; Johansen et al., 2022). No distinct characteristic, such as the inclusion of high-risk patients, the integration of medication reviews within the intervention or distinct post-discharge activities, distinguished successful from unsuccessful studies.

The inconsistent results described in our study, which showed a large effect of medication reconciliation on the reduction of clinically important medication errors but no effect on healthcare utilization, are disappointing. Indeed, healthcare utilization is an important outcome that needs to be considered when introducing new services. However, in addition to medication errors, healthcare utilization is influenced by numerous other factors, such as age, the number of medications taken at admission and the reason for admission, as in the present study. Furthermore, only a portion of healthcare utilization is medication related (Ravn-Nielsen et al., 2018), and only a portion is preventable (Jencks, Williams, and Coleman, 2009; van Walraven, 2014). For example, it is estimated that only one in five 30-day readmissions is medication related (Ravn-Nielsen et al., 2018) and that only 40% of these readmissions could be prevented (van Walraven, 2014; El Morabet et al., 2018; Meurs et al., 2021; Uitvlugt et al., 2022). Therefore, medication reconciliation interventions may only partially change overall healthcare utilization, even if they focus on more stringent outcomes such as medication-related hospitalizations.

Nevertheless, our results clearly showed that the medication reconciliation service, as provided in the current study and within routine clinical practice, was effective at reducing the occurrence of clinically important medication errors at hospital discharge. The high rate of more than 60% of patients with clinically important medication errors in the control group, with some patients being discharged with up to 10 clinically important medication errors, requires, in our opinion, the implementation of medication reconciliation as a fundamental, albeit not sufficient, element to ensure patient safety. Finally, we believe that the insights gained in our study can significantly contribute to the development, implementation and delivery of seamless care on a national level. This contribution becomes even more crucial following the national reimbursement of pharmacist-led seamless care programs in 2023.

### 4.1 Limitations and strengths

One of the major strengths of our study is that we evaluated the benefits of a pharmacist-led medication reconciliation intervention in routine clinical practice. The high rate of correction of medication errors at discharge in the intervention group suggested good integration of the service into ward routines, although this was not formally assessed. As we have described in our previous research (Knez et al., 2011a; Jošt et al., 2022), the integration of new pharmacy services can be challenging (Schnipper et al., 2022), particularly in settings that have only recently introduced clinical pharmacy. As most of the research on medication reconciliation comes from countries with a long tradition of clinical pharmacy (Anderson et al., 2019), our findings should be very informative for many settings in Central-Eastern Europe. The pragmatic design of our trial with broad patient inclusion criteria (Zwarenstein et al., 2008) allowed the inclusion of patients who are usually excluded from studies evaluating pharmacist-led interventions (Ravn-Nielsen et al., 2018; Graabaek et al., 2019; Karapinar-Çarkıt et al., 2019; Kempen et al., 2021; Johansen, Halvorsen, Svendsen, et al., 2022), thus providing evidence of the benefits of medication reconciliation for the general population of hospitalized medical patients. Notably, outcome assessment was performed by independent observers who were not included in the service provision. Although the observers were not blinded to patient allocation, they were trained according to standard operating procedures to minimize the risk of bias.

The lack of randomization is an important limitation of the present study and was dictated by its primary aim. Specifically, our aim was to assess the benefit of medication reconciliation conducted as part of routine clinical practice. Thus, randomization at the patient and cluster levels could not be performed because it would lead to cross-contamination and inability to integrate services into routine clinical practice, respectively. Although the allocation of patients to wards was random *per se*, as it depended primarily on bed availability and was therefore not influenced by the conduct of this study, the lack of randomization may have led to bias. As measured biases, e.g., in baseline patient characteristics, were accounted for by conducting multivariable analyses, unmeasured bias due to differences in ward practices beyond the provision of medication reconciliation could not be evaluated. Additionally, as mentioned above, our study was not sufficiently powered for the primary outcome of unplanned healthcare utilization because of premature termination of patient recruitment due to the COVID-19 epidemic. The effect of medication reconciliation on overall healthcare utilization was probably overestimated because studies with larger effects were selected for sample size calculations. Nonetheless, this study demonstrated the high validity of medication reconciliation, as carried out in our study, which is a prerequisite for its implementation in more complex, interprofessional and transmural interventions to further improve patient safety.

## 5 Conclusion

This pragmatic trial confirmed that pharmacist-led medication reconciliation reduced the risk of clinically important medication errors at hospital discharge by 20-fold. Notably, this effect was achieved while providing medication reconciliation within routine clinical practice and in a country where clinical pharmacy services are relatively new, in contrast to countries with long-standing tradition of clinical pharmacy. However, the provided service did not lead to a reduction in healthcare utilization within 30 days of discharge. Since various factors beyond medication errors contribute to post-discharge healthcare utilization, the medication reconciliation process employed in this study should be regarded as a crucial, but not sufficient, element to guarantee patient safety.

## Data Availability

The original contributions presented in the study are included in the article/Supplementary Material, further inquiries can be directed to the corresponding author.
