# Supplementary material for: Effectiveness of pharmacist-led medication reconciliation on medication errors at hospital discharge and healthcare utilization in the next 30 days: a pragmatic clinical trial
Source: Front Pharmacol. 2024 Mar 28;15:1377781. doi: 10.3389/fphar.2024.1377781 (PMC11007427; doi:10.3389/fphar.2024.1377781)
Supplement: Supplementary file 1 [file Table1.pdf]

Supplementary Table 1: Examples of clinically important medication errors at hospital discharge

Medication details in:		Description of medication error
**Best possible medication history**	**Discharge letter**	
Short acting insulin 6 iu + 4 iu + 4 iu sc inj, with meals	Omitted	In the discharge letter, insulin (or any other antidiabetic) therapy was omitted, despite the patient was receiving short acting insulin during hospitalisation. The medicine was not mentioned and no reason was given.
Rivaroxaban 20 mg tbl po od	Omitted	In the discharge therapy, rivaroxaban was omitted and acetylsalicylic acid (100 mg od) was introduced. The indication for rivaroxaban prior to hospitalisation was stroke prevention in atrial fibrillation, the patient had already experienced a past CVI. Rivaroxaban was not mentioned and no reason for omission was given.
Furosemide 40 - 80 mg tbl po od, in the morning	Furosemide 250 mg tbl – 1/2 tbl od, in the morning	In the discharge letter, furosemide 250 mg tbl – 1/2 tbl od, in the morning was directed. However, oral furosemide is only available in 40 and 500 mg strengths. Also, the dose of furosemide was increased. The instructions were not clearly written nor was the increase explained. The patient was admitted for pleural effusion and heart failure, had concurrent chronic renal failure and pneumonia, posing him to a risk of dehydration or heart failure decompensation.
Tacrolimus 2,5 mg tbl po od, after lunch	Tacrolimus 3,5 mg tbl po od, after lunch	In the discharge letter, tacrolimus dose was increased and no reason was given.
Acetylsalicylic acid 100 mg GR tbl po od	Omitted	In the discharge therapy, acetylsalicylic acid was omitted. Prior hospitalisation, the patient was taking also dabigatran (110 mg cps po bid), which was continued. The indications for anticoagulation and antiplatelet therapy were past MI and atrial fibrillation. No reason for omission was given.
Nebivolol 5 mg tbl po od	Omitted	In the discharge letter, nebivolol was omitted and bisoprolol (2,5 mg tbl po od) was introduced. The medicine was not mentioned and no reason was given.
Bisoprolol 1,25 mg tbl po od	Omitted	In the discharge letter, bisoprolol was not mentioned and amiodarone (200 mg tbl po od) was introduced. No reason for the change and no prescription for amiodarone was given.
Theophylline 350 mg cps po bid	Omitted	In the discharge letter, theophylline was omitted. No reason for omission of the drug with a narrow therapeutic window, previously prescribed in a high dose, was given.
No digoxin before admission	Methyldigoxin 0,1 mg tbl po od	In the discharge letter, methyldigoxin was prescribed. However, no reason for the introduction of the drug with a narrow therapeutic window was given.
Diclofenac 75 mg GR, SR cps po prn	Omitted	In the discharge therapy, diclofenac was not mentioned and it was not recommended to avoid it, although warfarin was introduced in addition to acetylsalicylic acid in patient with oesophageal varices.
Pantoprazole 40 mg GR tbl po prn	Omitted	In the discharge therapy, pantoprazole was omitted, moreover warfarin was introduced in addition to acetylsalicylic acid therapy. The indication for pantoprazole were varices of oesophagus.
